# A new species and two new records of *Goniothalamus* (Annonaceae) from Lao PDR

**DOI:** 10.3897/phytokeys.138.38995

**Published:** 2020-01-10

**Authors:** Bin Yang, Ren-Bin Zhu, Hong-Bo Ding, Somsanith Bouamanivong, Yun-Hong Tan

**Affiliations:** 1 Southeast Asia Biodiversity Research Institute, Chinese Academy of Sciences & Center for Integrative Conservation, Xishuangbanna Tropical Botanical Garden, Chinese Academy of Sciences, Menglun, Mengla, Yunnan 666303, China; 2 Center of Conservation Biology, Core Botanical Gardens, Chinese Academy of Sciences, Menglun, Mengla, Yunnan 666303, China; 3 Chinese Union of Botanical Gardens Secretariat, Xishuangbanna Tropical Botanical Garden, Chinese Academy of Sciences, Menglun, Mengla, Yunnan 666303, China; 4 Ecology Division, Biotechnology and Ecology Institute, Ministry of Science and Technology, P.O. Box: 2279, Vientiane Capital, Lao PDR

**Keywords:** *
Goniothalamus
*, Laos, field survey, new species, new record

## Abstract

*Goniothalamus saccopetaloides* Y.H. Tan & Bin Yang, a new species is described and illustrated from Laos. This species shows morphological similarities to *G. yunnanensis* W.T. Wang, but it differs from the latter by having almost fleshy, involute and saccate outer petals, subglobose monocarps, and single seeded monocarps. *Goniothalamus cheliensis*, and *G. calvicarpus* are new records for the Flora of Lao PDR. A key to *Goniothalamus* species indigenous to Laos is provided here.

## Introduction

*Goniothalamus* (Blume) Hook.f. & Thomson, comprising over 130 species of trees and shrubs, are one of the largest palaeotropical genera in the Annonaceae, mainly distributed from India and Sri Lanka to tropical Australia and the South Pacific Islands (Saunders and Chalermglin 2008; Nakkuntod et al. 2009; Turner 2014; Thomas et al. 2017). The genus is characterized by flowers with two whorls of petals of which the inner petals are smaller than the outer ones; three inner petals form a distinctive mitriform dome over the reproductive organs acting as a pollination chamber (Saunders and Chalermglin 2008; Tang et al. 2015), and stamens with apical connectives. Although the genus *Mitrephora* J. D. Hooker & Thomson also shares these similar features, *Goniothalamus* can be distinguished easily by its linear-oblong stamens, inner petals each with a short claw or stipe (Li et al. 2011). The genus shows considerable diversity in flowers of different size and shape, hairy indumentum, and color usually cream, yellow or red at maturity, and fruit morphology, with fruit of different size and shape (Saunders and Chalermglin 2008; Tang 2014; Tang et al. 2015). Species of the genus are widely distributed in tropical South-East Asian lowland and submontane forests (Tang 2014). Several *Goniothalamus* species have been described in recent years, including four new species from Thailand (Saunders and Chalermglin 2008), four new species from Borneo (Turner and Saunders 2008), and a new species from Palawan, the Philippines (Tang et al. 2013). In Laos, *Goniothalamus* are represented by four species (Newman et al. 2007; Lee 2016), i.e., *Goniothalamus laoticus* (Finet & Gagnep.) Bân, *Goniothalamus repevensis* Pierre ex Finet & Gagnep., *Goniothalamus marcanii* Craib and *Goniothalamus saigonensis* Pierre ex Finet & Gagnep., whereas the latter two were respectively treated as synonyms of *Goniothalamus tamirensis* Pierre ex Finet & Gagnep. (Saunders and Chalermglin 2008) and *Goniothalamus gabriacianus* (Baill.) Ast (Li et al. 2011).

In recent years, the authors examined the flowering material of Annonaceae cultivated in the living collections of Xishuangbanna Tropical Botanical Garden (XTBG), Chinese Academy of Sciences (CAS) and found an unknown species, which is very distinctive and belongs to the genus *Goniothalamus*; the records showed that this accession was originally collected in 2002, from Lao PDR (although the exact location remains unknown). Based on the morphological characters, we compared it to all other currently known species and concluded that it is new to science. As part of the botanical inventory of China-Laos transboundary biodiversity conservation, we carried out floristic surveys in the Nam Ha National Biodiversity Conservation Area in Luang Namtha Province and Phou Hin Phee National Biodiversity Conservation Area in Oudomxay Province of the northern Laos. During the fieldwork in March and October of 2018, we encountered two species of *Goniothalamus* representing new records for the flora of Laos. Therefore, the new and noteworthy species of *Goniothalamus* from Laos are provided and updated in this study.

## Material and methods

Our study of the new taxon was predominantly based on plant material newly collected in XTBG. The records showed that this accession was originally collected in 2002, from Laos, although the exact location remains unknown. Ten individuals cultivated in two living collections were observed. Morphological characterizations were measured in the field. We compared our samples with type specimens of similar species deposited in herbaria. Specimens of *Goniothalamus* from Laos and neighboring regions were examined from the following herbaria: HITBC, IBSC, SING, A, K, BK, high-resolution digital images of specimens (especially types) from JSTOR Global Plants (https://plants.jstor.org/) and other virtual herbarium websites (http://www.cvh.ac.cn/), as well as the taxonomic literature for species identification.

## Taxonomic treatments

### Goniothalamus saccopetaloides

Taxon classificationPlantaeMagnolialesAnnonaceae

Y.H.Tan & Bin Yang sp. nov.

56536A30-729B-51A7-9331-CB3A06E284C4

urn:lsid:ipni.org:names:77204192-1

Fig. 1

#### Diagnosis.

*Goniothalamus saccopetaloides* is morphologically similar to *G. yunnanensis* W.T. Wang with elliptic-oblong to oblong leaf blades and broadly lanceolate, pinkish orange to reddish brown outer petals, but easily distinguished by having almost fleshy, involute, saccate outer petals, subglobose and single seeded monocarps.

#### Type.

Lao PDR. Specific location unknown. Voucher from a cultivated plant at the Xishuangbanna Tropical Botanical Garden, Chinese Academy of Sciences, 2 May 2019, *B. Yang*, *XTBG-0054* (holotype, HITBC!).

#### Description.

Shrub to small tree, to 3 m tall. Young branches glabrous. Leaf laminas 15.5–27.8 cm long, 4.5–8.2 cm wide, length/width ratio ca. 3.4, elliptic-oblong to oblong, apex acuminate, base cuneate to attenuate, papyraceous, glabrous abaxially and adaxially; midrib glabrous and (strong) prominent abaxially, glabrous and impressed adaxially; secondary veins 13–18 pairs, (slightly) impressed adaxially; tertiary veins percurrent, distinct; petioles 5–12 mm long, 2–3 mm in diameter, glabrous. Flowers 1–7, often on the main trunk (cauliflory) and on older branches (ramiflory), pendents; flowering pedicels 2–5 mm long, sparsely hairy; pedicel bracts 3–6, 1–2 mm long. Sepals 5–6 mm long, 5–7 mm wide, basally connate, apex acuminate, broad ovate, puberulous abaxially, glabrous adaxially, greenish-yellow, venation longitudinal slightly conspicuous adaxially, indistinct abaxially. Outer petals 1.4–2.7 cm long, 0.7–1.2 mm wide, length/width ratio 1.6–3.0, almost fleshy, involute, saccate, elliptic lanceolate to broadly lanceolate, sparsely puberulous abaxially, subglabrous adaxially, yellowish green in young stage, pinkish orange to reddish brown in mature stage, venation slightly distinct adaxially, occasionally slightly distinct abaxially. Inner petals 6.5–12 mm long, 5–10 mm wide, length/width ratio 1.1–2.2, broadly ovate to obovate, sparsely puberulous abaxially, pubescent adaxially, yellowish green in young stage, pinkish orange to reddish brown in mature stage, base attenuate to a 2–3 mm claw. Stamens ca. 80 per flower, 1.2–1.5 mm long, 0.6–0.7 mm wide; connectives truncate. Carpels 18–22 per flower, ovary 1.5–2 mm long, light green, with white hairs; stigma and style 2–3 mm long, glabrous. Immature fruits green, with small white dots, mature fruits orange to red; fruiting pedicels 3–5 mm long, 1.5–2 mm in diameter, subglabrous. Monocarps one seeded, 12–13 mm long, 9–11 mm wide, length/width ratio 1.2–1.3, subglobose to ellipsoid, base and apex rounded, smooth, subglabrous, glossy, pericarp medium-thick, ca. 1.5 mm thick, stipes subsessile to 1.5 mm long, ca. 2 mm in diameter, glabrous. Seeds 9–10 mm long, ca. 7 mm wide, length/width ratio 1.3–1.4, ellipsoids, testa slightly rugose, dark brown to black brown, aril orange.

**Figure 1.**
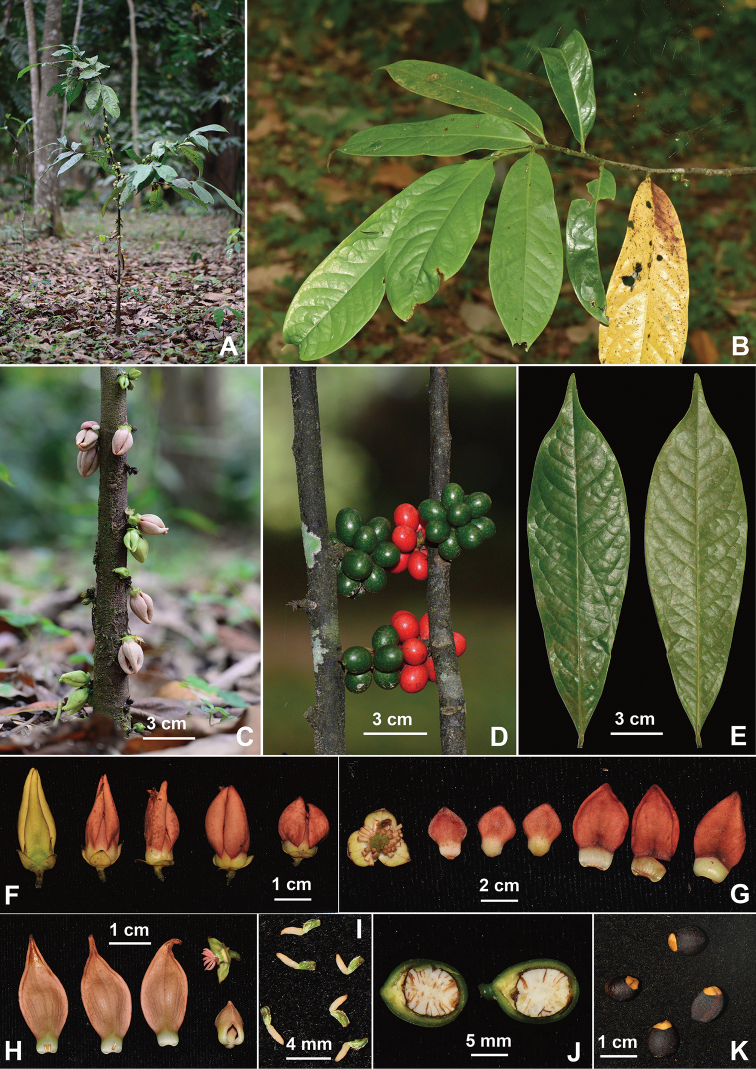
*Goniothalamus saccopetaloides* Y.H.Tan & Bin Yang, sp. nov. **A** habit **B** leafy branch **C** flowers on main trunk **D** fruits on main trunk **E** leaves (adaxial and abaxial surface) **F** flowers **G–H** dissected flowers **I** carpels **J** longitudinally dissected monocarp **K** seeds. Photographed by R.B. Zhu and B. Yang.

#### Etymology.

The new species is named after its saccate and almost fleshy outer petals.

#### Phenology.

*Goniothalamus saccopetaloides* has been observed in flowers from the end of March to May and in fruits from July to September.

#### Distribution and habitat.

*G. saccopetaloides* was originally distributed in Laos; the exact location and its wild habitat remains unknown. Additional collections in the future may help to clarify its full distribution.

#### Conservation status.

Due to insufficient field surveys so far, very limited details about its natural distribution and population status are currently known. Further investigation is required to determine more distribution sites and conservation status of the new species; at this moment we consider it as data deficient (DD) according to the IUCN Red List Categories (IUCN 2012).

#### Additional specimen examined

(paratype). Lao PDR. Specific location unknown. Voucher from the cultivated plants at the Xishuangbanna Tropical Botanical Garden, Chinese Academy of Sciences, 2 May 2019, *B. Yang*, *XTBG-0055* (HITBC!).

#### Notes.

*Goniothalamus saccopetaloides* is morphologically similar to *G. yunnanensis*; however, in addition to the description in the diagnosis, the two species can be easily distinguished by other characters. *Goniothalamus saccopetaloides* has 1 to 7 flowers, often on the main trunk (cauliflory) and on older branches (ramiflory), scarcely on young growth, whereas *G. yunnanensis* has 1 to 2 flowers, axillary, often on young growth, sometimes from leafless nodes (Li et al. 2011). Moreover, *G. saccopetaloides* has one ovule per carpel, subglobose monocarps and seeds with rounded apices, and *G. yunnanensis* has 2 ovules per carpel, ellipsoid monocarps and seeds with acute apices (Li et al. 2011).

### New records for Laos

#### Goniothalamus calvicarpus

Taxon classificationPlantaeMagnolialesAnnonaceae

Craib

C7098EDB-8FF0-5F9D-BFAE-BD155DF41F26

Fig. 2 (A–D)

Goniothalamus calvicarpus Craib, Bull. Misc. Inform. Kew. 1922: 227 (1922). – TYPE: Thailand: Sukotai, Kao Luang, Sukhothai Province, Northern Thailand, 4 May 1922, *A.F.G. Kerr 5946* (holotype: K!; isotypes: BK!, BM!).

##### Distribution and habitat.

Thailand (Saunders and Chalermglin 2008), China (Li et al. 2011) and Northern Laos (Luang Namtha Province), mountain slopes, 800–1500 m.

##### Specimens examined.

Laos: Tha Se Village, Nam Ha National Biodiversity Conservation Area, Luang Namtha Province, Northern Laos, tropical lower montane forest, 938 m, 20°49'59.42"N, 101°15'48.05"E, 21 Oct 2018, *Y.H. Tan et al.*, *L0757* (HITBC); idem, 21 Oct 2018, *Y.H. Tan et al.*, *L0764* (HITBC); idem, 26 Mar 2018, *Y.H. Tan et al.*, *L0183* (HITBC); Nam Sing Village, Nam Ha National Biodiversity Conservation Area, Luang Namtha Province, Northern Laos, stone forest, 900 m, 20°45'09"N, 101°12'08"E, 25 Mar 2018, *Y.H. Tan et al.*, *L0153* (HITBC).

**Figure 2.**
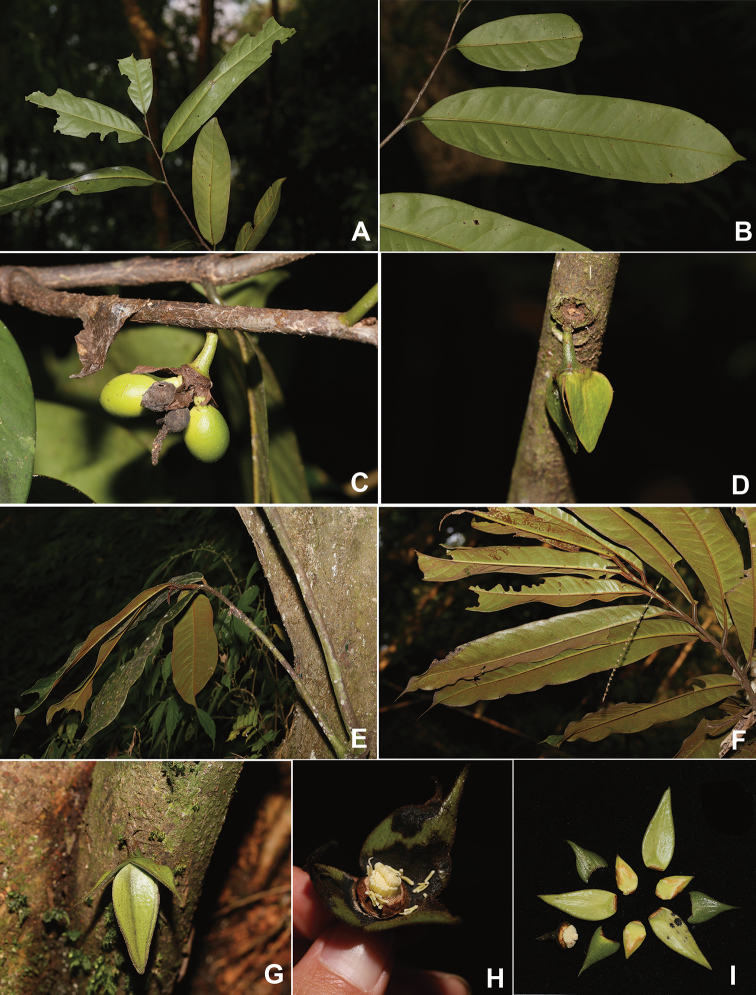
*Goniothalamus calvicarpu*s Craib **A** leafy branch **B** leaves (abaxial) **C** fruit **D** flower (epibiotic sepels); *Goniothalamus cheliensis* Hu **E** leafy branch **F** leaves (abaxial) **G** flower **H** flower (picked petals, showing stamens and carpels) **I** dissected flowers. Photographed by H.B. Ding.

##### Notes.

There has been considerable taxonomic confusion between *G. calvicarpus* and *G. griffithii* Hook. f. & Thomson (Saunders and Chalermglin 2008), the Chinese material identified as *G. griffithii* to be more correctly placed in *G. calvicarpus* (Saunders and Chalermglin 2008; Li et al. 2011). *Goniothalamus calvicarpus* is relatively widely distributed in the lower mountain evergreen forest in Xishuangbanna, southern Yunnan, compared with our materials collected in Northern Laos, the characters of flowers and fruits are exactly in line with *G. calvicarpus*; it is also not very hard to encounter around the adjacent areas in Northern Laos bordering southern Yunnan.

#### Goniothalamus cheliensis

Taxon classificationPlantaeMagnolialesAnnonaceae

Hu

CC3F30EC-3E58-5C86-8E4A-2A919DEC96EA

Fig. 2 (E–I)

Goniothalamus cheliensis Hu, Bull. Fan Mem. Inst. Biol. 10: 122 (1940). – TYPE: China: Che-Li Hsien, Maan-Shang, Yunnan, Sep. 1936, *C.W. Wang 78573* (holotype: TAI!, isotypes: A!, IBSC!].

##### Distribution and habitat.

Northern Thailand (Saunders and Chalermglin 2008), China (Li et al. 2011) and Northern Laos (Oudomxay Province), Montane forests; 1000–1500 m.

##### Specimens examined.

Laos: Maung Xai, Phou Hin Phee National Biodiversity Conservation Area, Oudomxay province, 1357 m, 20°43'19.12"N, 102°08'46.61"E, 30 March 2018, *Y.H. Tan et al.*, *L0350* (HITBC); ibdem, 1372 m, 20°43'18.24"N, 102°08'47.31"E, 30 March 2018, *Y.H. Tan et al.*, *L0351* (HITBC).

##### Notes.

*Goniothalamus cheliensis* is a very distinctive species, with leaves, flowers and fruits very large and densely rust-colored hirsute (Saunders and Chalermglin 2008), according to our personal observation, the monocarps of *G. cheliensis* can be up to 15 cm long.

With the addition of *Goniothalamus saccopetaloides*, *Goniothalamus cheliensis*, and *Goniothalamus calvicarpus*, seven species are currently recognized in Laos (Newman et al. 2007; Saunders and Chalermglin 2008; Li et al. 2011; Lee 2016). A key is provided below to further elucidate the morphological differences among the species occurring in Laos.

###### Key to the *Goniothalamus* species in Laos and along with *G. yunnanensis*

**Table:** 

1	Young branches densely hairy to velutinous, leaf midrib and petiole (very densely) hairy abaxially	**2**
–	Young branches glabrous, leaf midrib and petiole glabrous to sparsely hairy abaxially	**3**
2	Leaf blade 50–76 × 13–22 cm, stamen connectives apiculate, monocarps oblong, 3–15 cm long and densely hairy	***G. cheliensis***
–	Leaf blade 12–17 × 3.7–4.7 cm, stamen connectives truncate, monocarps ovoid, less than 3 cm long and glabrous	***G. tamirensis***
3	Adaxial surface of leaves with very prominent secondary and tertiary veins	**4**
–	Adaxial surface of leaves with impressed or only slightly prominent secondary and tertiary veins	**6**
4	Stamen connectives apiculate, sepals 11–18.5 mm long	***G. calvicarpus***
–	Stamen connectives truncate, sepals 5–8 mm long	**5**
5	Flowers on older branches and young growth, outer petals not fleshy, never involute, monocarps ellipsoid, 1.8–2.5 cm long, apex acute	***G. yunnanensis***
–	Flowers on main trunk and older branches, outer petals almost fleshy, involute, monocarps subglobose to ellipsoid, 1.2–1.3 cm long, apex rounded	***G. saccopetaloides***
6	Ovules 8–10 per carpel, monocarps oblate, with its longitudinal ridge	***G. laoticus***
–	Ovules 1–2 per carpel, monocarps oblong-ellipsoid to ellipsoid, or elliptic-ovoid	**7**
7	Leaf blades with short to long acuminate apices, outer petal venation distinct, sparsely puberulous to glabrous abaxially	***G. repevensis***
–	Leaf blades with acute to attenuate apices, outer petal venation indistinct, hispidulous abaxially	***G. gabriacianus***

## Supplementary Material

XML Treatment for Goniothalamus saccopetaloides

XML Treatment for Goniothalamus calvicarpus

XML Treatment for Goniothalamus cheliensis
